# TLR-4 ligation of dendritic cells is sufficient to drive pathogenic T cell function in experimental autoimmune encephalomyelitis

**DOI:** 10.1186/1742-2094-9-248

**Published:** 2012-10-30

**Authors:** Richard J Mellanby, Helen Cambrook, Darryl G Turner, Richard A O’Connor, Melanie D Leech, Florian C Kurschus, Andrew S MacDonald, Bernd Arnold, Stephen M Anderton

**Affiliations:** 1 Medical Research Council/University of Edinburgh Centre for Inflammation Research, Centre for Multiple Sclerosis Research and Centre for Immunity Infection and Evolution, Queen’s Medical Research Institute, 47 Little France Crescent, Edinburgh, EH16 4TJ, UK; 2 Institute for Molecular Medicine, University Medical Center of the Johannes Gutenberg University, Mainz, 55131, Germany; 3 Institute of Immunology and Infection Research, University of Edinburgh, Edinburgh, EH9 3JT, UK; 4 Deutsches Krebsforschungszentrum DKFZ, Molekulare Immunologie, Im Neuenheimer Feld 280, Heidelberg, 69120, Germany

**Keywords:** Multiple sclerosis, Experimental autoimmune encephalomyelitis, Dendritic cells, Myelin basic protein

## Abstract

**Background:**

Experimental autoimmune encephalomyelitis (EAE) depends on the initial activation of CD4^+^ T cells responsive to myelin autoantigens. The key antigen presenting cell (APC) population that drives the activation of naïve T cells most efficiently is the dendritic cell (DC). As such, we should be able to trigger EAE by transfer of DC that can present the relevant autoantigen(s). Despite some sporadic reports, however, models of DC-driven EAE have not been widely adopted. We sought to test the feasibility of this approach and whether activation of the DC by toll-like receptor (TLR)-4 ligation was a sufficient stimulus to drive EAE.

**Findings:**

Host mice were seeded with myelin basic protein (MBP)-reactive CD4+ T cells and then were injected with DC that could present the relevant MBP peptide which had been exposed to lipopolysaccharide as a TLR-4 agonist. We found that this approach induced robust clinical signs of EAE.

**Conclusions:**

DC are sufficient as APC to effectively drive the differentiation of naïve myelin-responsive T cells into autoaggressive effector T cells. TLR-4-stimulation can activate the DC sufficiently to deliver the signals required to drive the pathogenic function of the T cell. These models will allow the dissection of the molecular requirements of the initial DC-T cell interaction in the lymphoid organs that ultimately leads to autoimmune pathology in the central nervous system.

## Findings

The clinical manifestation of experimental autoimmune encephalomyelitis (EAE) depends on the initial activation of CD4^+^ T cells responsive to myelin autoantigens and their differentiation into autoaggressive effector T cells which, when re-isolated from the inflamed central nervous system (CNS) can produce a range of pro-inflammatory cytokines including IFN-γ, IL-17, TNF-α and granulocyte-macrophage colony-stimulating factor (GM-CSF) [1]. The immunological consensus is that the key antigen presenting cell (APC) population, capable of driving the activation of naïve T cells most efficiently, is the dendritic cell (DC) [2]. As such, many studies have shown that inoculation of mice with antigen-loaded DC can substitute for active immunization with the antigen in adjuvant to provoke a T cell response. Achieving this as a robust experimental option for the induction of EAE would allow us to probe the molecular requirements for the initial DC-T cell interaction in the lymphoid organs that ultimately leads to autoimmune pathology in the CNS. It is notable, however, that despite some sporadic reports [3-5], this has not as yet been achieved. Classically, the induction of EAE requires the use of complete Freund’s adjuvant (CFA), which contains a range of mycobacterial-derived molecules to trigger DC activation through their pattern-recognition receptors (PRRs). The most common means of activating DC prior to their use for cellular immunization is to use the toll-like receptor (TLR)-4 agonist lipopolysaccharide (LPS). Thus, one possible reason for the lack of a robust DC-driven model of EAE may be that LPS-activated DC cannot provide all of the signals to the naïve myelin-response T cells to engender strong autoaggressive function.

To formally test this possibility, we sought to induce EAE using bone marrow-derived DC (BMDC) that had received only the TLR4-signal *in vitro*, by activation with LPS. To maximize our chances of success we first sought to obviate two possible confounding issues – the size of the naïve myelin-responsive repertoire and the ability of the DC to present the autoantigen. We therefore started by developing a transgenic two-cell transfer model. We have previously reported that C57BL/6xB10.PL mice are resistant to EAE induction using the Ac1-9 peptide of myelin basic protein (MBP), unless they are first seeded with a cohort of naïve MBP-responsive T cells derived from the Tg4 T cell receptor (TCR) transgenic mouse [6]. This approach therefore allowed us to control the size of the Ac1-9-responsive T cell repertoire. To remove the possibility that BMDC may for some reason fail to present the Ac1-9 peptide efficiently, we made use of a second transgenic line, the AMK_35_ mouse, in which major histocompatibility molecule (MHC) class II-expressing cells constitutively express the Ac1-9 peptide, covalently bound to the A^U^ molecule [7].

**Figure 1:**
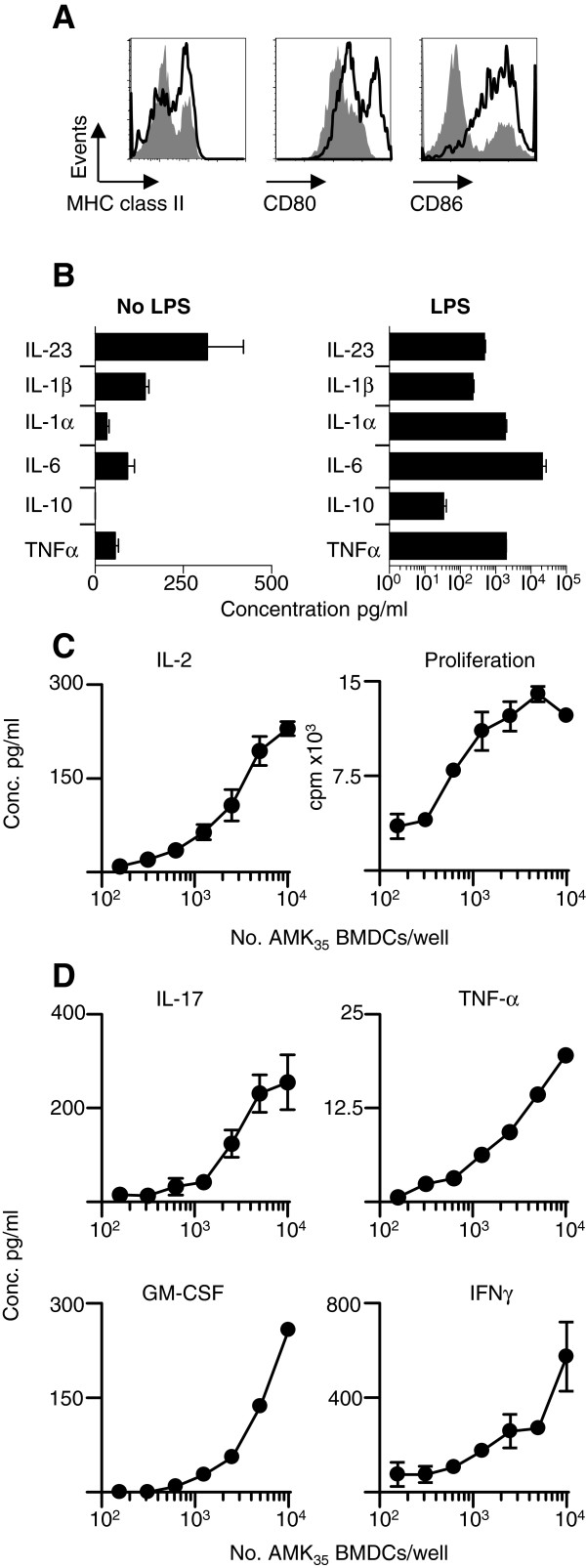
**MBP loaded BMDC can prime MBP responsive T cells.** BMDC were generated in the presence of 20ng/ml of recombinant GM-CSF for nine days as previously described [11] and activated with 0.1μg/ml LPS and 5ng/ml of GM-CSF for an additional 18 hours. **A**) Cells were stained for MHC class II, CD80, CD86 expression with (open histogram) or without (grey histogram) LPS maturation and analyzed by FACS. **B**) Cytokine concentrations in BMDC supernatants were sampled after 18-hour culture with or without LPS. **C** and **D**) To study the primary activation of Tg4 T cells, varying numbers (as stated) of AMK_35_[7] BMDC were cultured with 2x10^4^ CD4^+^ Tg4.CD45.1+ T cells [12] per well. Cell proliferation was assessed by thymidine incorporation. The results are expressed as mean counts per minute ± standard error of the mean. Tg4 T cell production of cytokines (IL-2, IL-17A, GM-CSF, TNF-α and IFN-γ) was assessed in culture supernatants by ELISA. IL-2 was measured in supernatants after 48 hours of culture and IFN-γ, TNF-α, GM-CSF and IL-17A were measured after 72 hours of culture. Data are representative of four independent experiments. BMDC, bone marrow-derived dendritic cells;FACS, fluorescence activated cell sorting; GM-CSF, granulocyte-macrophage colony-stimulating factor; LPS, lipopolysaccharide; MBP, myelin basic protein.

**Figure 2:**
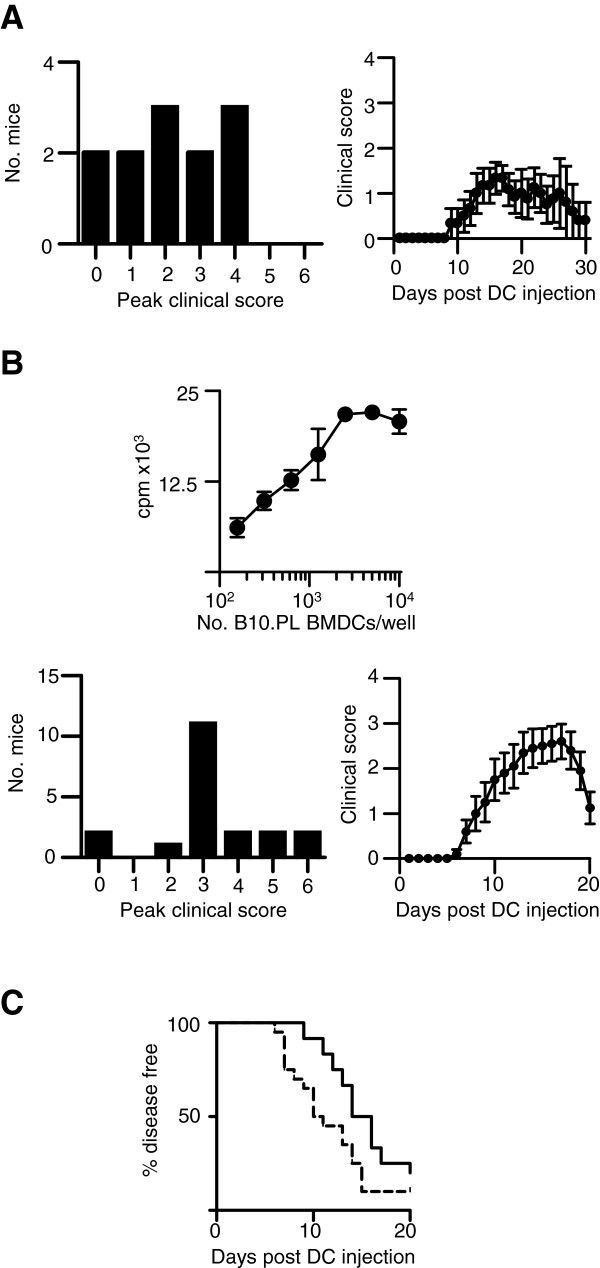
**MBP loaded BMDC can initiate EAE in mice seeded with MBP-reactive T cells.** BMDC were generated from AMK_35_ or B10.PL mice and matured with LPS as described in Figure 1. A total of 2x10^6^ Tg4.CD45.1 CD4+ T cells was transferred into B10.PLxC57BL/6 mice one day prior to s.c. injection of 2x10^6^ LPS-conditioned AMK_35_ BMDC (**A**) or B10.PL BMDC pulsed with 0.1μM MBP Ac1-9(4Tyr) analog peptide (**B**). of Pertussis toxin was administered (200 ngi.p.) at the time of BMDC transfer. Clinical signs of EAE were assessed daily with the following scoring system: 0, no signs; 1, flaccid tail; 2, impaired righting reflex and/or gait; 3, partial hind limb paralysis; 4, total hind limb paralysis; 5, hind limb paralysis with partial front limb paralysis; 6, moribund or dead. **A**) Plots show maximal clinical scores of mice administered AMK_35_ BMDC and the clinical disease curve of three pooled, independent experiments. **B**) A total of 2x10^4^ Tg4 CD4+ T cells was cultured with LPS-conditioned B10.PL BMDC pulsed with 0.1μM MBP Ac1-9(4Tyr) and assessed for proliferation (thymidine incorporation). Plots show maximal clinical scores of mice administered B10.PL BMDC pulsed with 0.1μM MBP Ac1-9(4Tyr) and the clinical disease curve of three pooled, independent experiments. **C**) Plot showing timing of disease onset in mice administered AMK_35_ DC (black line, n=12) and MBP Ac1-9(4Tyr) pulsed DC (dashed line, n=20). All mice were bred under specific pathogen-free conditions at the University of Edinburgh and all experiments had local ethical approval and were performed in accordance with UK legislation. BMDC, bone marrow-derived dendritic cells; DC, dendritic cells; EAE, experimental autoimmune encephalomyelitis; LPS, lipopolysaccharide; MBP, myelin basic protein.

BMDC generated from AMK_35_ mice showed elevated expression of A^u^, CD80 and CD86 after overnight exposure to LPS (Figure 1A) and supernatants from these cultures contained elevated levels of pro-inflammatory cytokines (Figure 1B), notably IL-1β, IL-6 and IL-23, each of which has been described as being required for EAE development using gene-deficient mice [8-10]. These LPS-activated AMK_35_ DC also efficiently induced the *in vitro* proliferation of naïve Tg4 CD4^+^ T cells (Figure 1C), and their production of IFN-γ, IL-17, TNF-α and GM-CSF (Figure 1D), without any addition of exogenous antigen to the culture. Thus, LPS-activated DC appeared to have many of the key attributes associated with EAE pathology. To test this *in vivo*, we seeded C57BL/6xB10.PL mice with a cohort of naïve Tg4 CD4+ T cells and one-day later subcutaneously administered LPS-activated AMK_35_ DC. Ten out of 12 mice developed clinical signs of EAE (Figure 2A). However, the extent and, importantly, the timing of onset of clinical signs showed variation.

The use of BMDC derived from mice in which antigen presentation is driven by a transgene, of course, has limitations. For example, it can be envisaged that it would be desirable to study DC expressing multiple autoantigenic peptides, either separately or together. To extend our study in this direction we therefore generated BMDC from A^U+^ non-transgenic BMDC, from B10.PL mice. These BMDC were activated with LPS and loaded with the Ac1-9(4Tyr) peptide *in vitro*. This variant peptide binds to the A^u^ molecule with high affinity, allowing stable antigen presentation (unlike the wild type Ac1-9 peptide). These antigen-loaded non-transgenic DC could efficiently activate naïve Tg4 T cells *in vitro* (Figure 2B) and induced a robust disease course in the T cell/DC transfer model in C57BL/6xB10.PL mice (Figure 2B). Of note, there was a trend for the onset of disease to be earlier and more synchronous than achieved with AMK_35_ DC (Figure 2C).

The two novel models of DC-driven EAE we have described have a number of advantages over the few earlier reports of EAE following transfer of autoantigen-loaded DC. Previous studies using BMDC pulsed with the 35–55 peptide of myelin oligodendrocyte glycoprotein (MOG) in C57BL/6 mice have required repeated injections of BMDC to induce EAE [3] or have required concurrent administration of CFA to drive robust disease [5]. A previous report of EAE involving MBP-pulsed DC differed markedly from ours in that five times as many MBP-reactive T cells were transferred and the host mice were irradiated prior to cell transfer [4]. Of note, the wild type MBP peptide (lacking the insertion of a Tyr residue at position 4) has such low affinity for A^u^ that APC cannot be pulsed with that peptide to allow efficient loading into the A^u^ peptide binding groove [13]. It is therefore difficult to reconcile that observation with the reported ability of DC loaded with the wild type peptide to activate MBP-responsive T cells for the initiation of EAE [4]. Unlike that previous report, we used two systems in which the presentation of the autoantigenic peptide-MHC complex by the DC was highly stable. AMK_35_ DC, expressing transgenic peptide-MHC [7] induced variable degrees of EAE, particularly in terms of day of onset, but this difficulty was overcome using non-transgenic DC pulsed with the Ac1-9(4Tyr) peptide, which shows extraordinary affinity for the A^U^ molecule [14]. DC that are loaded with this peptide *in vivo* can maintain expression of the peptide-MHC complex for at least seven days (SMA, unpublished). Given that the consensus is that BMDC probably persist for only a few days after administration, we can be confident that the DC we transferred were presenting the peptide to T cells for a sustained length of time. It is conceivable that the MOG(35–55)-A^b^ complex might be prone to more rapid degradation in DC transfer experiments. Nevertheless, it is evident from other studies that the use of MOG(35–55)-loaded C57BL/6 DC can be sufficient to provide TCR-signaling *in vivo*, as we ourselves have shown [15,16], but it was not sufficient to trigger robust EAE, which still required subsequent immunization with CFA in those models.

A key paradigm is that autoreactive T cells can develop into autoaggressive T cells if they recognize (self, or cross-reactive non-self) antigen presented during infection (that is, on activated APC). The use of CFA to induce EAE mimics infection due to the presence of heat-killed mycobacteria. However, CFA contains multiple moieties capable of stimulating APC through a range of PRRs. Although LPS is not one of these moieties, heat shock proteins 65 and 70 have been described as TLR-4 agonists [17], as has pertussis toxin [18], which is routinely used during EAE induction. Less complex adjuvants, including LPS, have been reported to be able to substitute for CFA [19-22], but results have been variable between studies. Indeed, exposure to LPS can protect against EAE if it occurs before immunization with autoantigen in CFA [23]. Those studies have not provided clear information on which APC need to be activated through a given PRR to drive pathology, or which of the multiple consequences of PRR-ligation are non-redundant for the induction of EAE. Furthermore, it is clear that expression of the same TLR on different immune cells can have markedly different effects on disease outcome [24,25].

From our experiments described here, we can conclude that a) DC are sufficient as APC to effectively drive the differentiation of naïve myelin-responsive T cells into autoaggressive effector T cells and b) exposure to a TLR-4 agonist can activate the DC sufficiently to deliver the signals required to drive the pathogenic function of the T cell. These new model systems will therefore allow comparison of a range of PRR stimuli upon the DC and the use of gene-knockout/knockdown DC to help to probe the key molecular events in the initial DC-T cell dialogue that lead ultimately to autoimmune CNS inflammation in EAE.

## Abbreviations

APC: Antigen presenting cell; BMDC: Bone marrow derived dendritic cell; CFA: Complete Freund’s adjuvant; CNS: Central nervous system; DC: Dendritic cell; EAE: Experimental autoimmune encephalomyelitis; ELISA: Enzyme-linked immunosorbent assay; FACS: Fluorescence activated cell sorting; GM-CSF: Granulocyte-macrophage colony-stimulating factor; IFN: Interferon; IL: Interleukin; LPS: Lipopolysaccharide; MBP: Myelin basic protein; MHC: Major histocompatibility molecules; MOG: Myelin oligodendrocyte glycoprotein; PRRs: Pattern-recognition receptors; TCR: T cell receptor; TLR: Toll-like receptor; TNF: Tumor necrosis factor; Tyr: Yrosine.

## Competing interests

The authors declare that they have no competing interests.

## Authors’ contributions

RJM, ASM and SMA designed the experiments. RJM, HC, DT, ROC and MDL performed the experiments. RJM and SMA drafted the manuscript. FCK and BA generated the AMK_35_ transgenic mouse. All authors have read and approved the final manuscript.
